# A Narrative Review of the Odyssey of Thyroid Cancer Diagnosis: Can 99mTc-SESTAMIBI Molecular Imaging Replace Fine Needle Aspiration Biopsy?

**DOI:** 10.3390/medicina61061043

**Published:** 2025-06-05

**Authors:** Ioannis Iakovou, Nikitas Papadopoulos, Paraskevi Exadaktylou, Christos Melidis, Georgia Koutsouki, Ilias Katsadouros, Savvas Frangos, Ioannis Koutelidakis, Kalliopi Kotsa, Evanthia Giannoula

**Affiliations:** 1 Second Academic Nuclear Medicine Department, Academic General Hospital “AHEPA”, Aristotle University of Thessaloniki, 54636 Thessaloniki, Greece; iiakovou@auth.gr (I.I.); n_papadopoulos@outlook.com (N.P.); koutsoukig@gmail.com (G.K.); hliask77@gmail.com (I.K.); eva_giann@hotmail.com (E.G.); 2 CAP Santé, Radiation Therapy, Clinique Maymard, 20200 Bastia, France; melichristos@hotmail.com; 3 milliVolt.eu (A Health Physics Company), 20200 Bastia, France; 4 Nuclear Medicine Department and Thyroid Cancer Clinic, Bank of Cyprus Oncology Center, 2404 Nicosia, Cyprus; savvas.frangos@bococ.org.cy; 5 Second Department of Surgery, General Hospital of Thessaloniki “G. Gennimatas”, School of Medicine, Aristotle University of Thessaloniki, 54635 Thessaloniki, Greece; iokoutel@gmail.com; 6 First Department of Internal Medicine, Academic General Hospital “AHEPA”, School of Medicine, Aristotle University of Thessaloniki, 54636 Thessaloniki, Greece; kkalli@auth.gr

**Keywords:** thyroid malignancy, Sestamibi scintigraphy, Fine Needle Aspiration Biopsy, Bethesda, nodule

## Abstract

*Background and Objectives:* Many diagnostic methods exist for identifying thyroid malignancy, but most of them resemble an odyssey, as the journey from palpating a nodule to receiving a definitive diagnose is often long and costly. The aim of the present study is to investigate the role of Sestamibi scintigraphy in the characterization of cytological indeterminate thyroid nodules. *Materials and Methods:* A focused literature review was conducted, emphasizing the comparison between Fine Needle Aspiration Biopsy (FNAB), the main diagnostic method for thyroid cancer, and Sestamibi. *Results:* It is widely accepted that Sestamibi is the primary alternative for patients with non-diagnostic FNAB. As shown in the literature, Sestamibi has a high negative predictive value in excluding thyroid malignancy. *Conclusions:* Much like Odysseus’ adventurous 10-year journey returning to Ithaca, the path to diagnosing thyroid cancer is not straightforward. Molecular imaging with 99mTc-Sestamibi may serve as a valuable adjunct in evaluating thyroid nodules with inconclusive cytological findings.

## 1. Introduction

The word odyssey has come to mean a journey of epic proportions, derived from Homer’s namesake epic poem. The *Odyssey* lyrically describes Odysseus’ decade-long journey, filled with adventure, that delayed his return to his beloved homeland, Ithaca. A definitive diagnosis of thyroid cancer involves a long journey—often filled with challenges, whether invasive or not—that can lead to pain, loss, and significant expense.

Globally, thyroid cancer is the most common type of endocrine malignancy, with two-thirds of cases being diagnosed before the age of 55 [1]. The odyssey begins when thyroid nodules are detected, either through palpation by the patient or a physician (2–6%), or via imaging (19–35%) [2]. These nodules always need further investigation, as about 7% to 15% are eventually found to be cancerous [3]. Evaluation includes a detailed personal medical history, comprehensive clinical examination, measurement of Thyroid Stimulating Hormone (TSH), and ultrasound (US) imaging. Depending on the US findings, the physician may proceed with either Fine Needle Aspiration Biopsy (FNAB) or patient monitoring, based on various published guidelines ([4,5], for example). FNAB has become the most reliable method for evaluating thyroid nodules and determining which patients should undergo surgery. Thyroid scintigraphy with 99mTc, 131I, or 123I assesses nodule functionality, especially in multinodular goiter, by identifying hypofunctioning nodules that require biopsy [6].

An extensive review of the literature was conducted, emphasizing the comparison between FNAB, the main diagnostic method of thyroid cancer, and Sestamibi scintigraphy.

## 2. The Cicones: Ultrasound

All patients with thyroid nodules should undergo an US examination. US is extremely useful for determining the size and anatomy of the thyroid gland, as well as the surrounding neck structures. The likelihood of malignancy has been linked to specific ultrasound features, including solid composition, small volume, greater height than width, irregular margins, or evidence of extrathyroid expansion [7].

US, like the Ciconians who killed most of Odysseus’ weaker men but failed to defeat the strong ones, performs an initial sorting process—acting as a filter to identify nodules that are highly suspicious for malignancy.

Since 2009, classification systems have been developed to assess the risk of thyroid malignancy based on ultrasound characteristics, in order to distinguish patients who should undergo FNAB from those who should require monitoring. In 2012, the American College of Radiology proposed the Thyroid Imaging Reporting and Data System (TI-RADS), a grading and classification system for thyroid nodules based on US features, which indicates associated risk of malignancy [4]. Similarly, in 2017 the European Thyroid Association published equivalent guidelines for adults [EU-TIRADS] [5]. Although these US-based risk stratification systems (RSS) display high diagnostic accuracy, each with its own advantages and disadvantages, significant differences exist among them. EU-TIRADS shows a higher sensitivity, whereas the American College of Radiology TIRADS [ACR-TIRADS] has a higher specificity. Therefore, this presents a gap in which a significant number of cases cannot be clearly determined as malignant or benign. A table summarizing the sonographic features and their respective risk of malignancy is provided at the end of the text (Table 1).

## 3. Polyphemus the Cyclops: Fine Needle Aspiration Biopsy

After sailing calmly for many weeks, Odysseus and his men came ashore in the land of the Cyclopes, fierce giants with one eye in the middle of their foreheads.

Nodules suspected of malignancy end up in the ‘land of FNAB’. FNAB, currently considered the most reliable method for evaluating thyroid nodules, has minimal risk of complications. With a high positive predictive value of more than 97%, FNAB plays a crucial role in guiding optimal clinical and surgical management [9], thereby increasing the rate of thyroid cancers diagnosed pre-operatively. It answers the key question of diagnostic approach: surgery or surveillance? Nevertheless, FNAB is an invasive method and can not only result in false negatives due to low-quality aspirated material [10], but more importantly, it often leads to clinical dilemmas, as the management of patients with doubtful/intermediate cytological results remains a controversial subject.

As Fine Needle Aspiration Biopsy (FNAB) is the main diagnostic method for thyroid nodules and should be performed under ultrasound guidance, a combined assessment including laboratory findings and ultrasound-based risk stratification is recommended. A table at the end of the text outlines the Bethesda system classification and its associated likelihood of malignancy (Table 2). The nodule size combined with the EU-TIRADS classification system are factors that indicate FNAB. Therefore, FNAB is advised in cases, such as high-risk EU-TIRADS 5 with nodules >10 mm, intermediate-risk EU-TIRADS 4 with nodules >15 mm, and EU-TIRADS 3 (low risk) with nodules >20 mm.

More specifically, FNAB has low accuracy when differentially diagnosing the second most-common type of thyroid malignancy [11]—follicular cancer [12]. Thyroid nodules with an uncertain malignant potential are categorized as “Bethesda III” or “IV” [13], with the probability of malignancy being from 10% to 30% and from 25% to 40%, respectively. For these nodules, it is recommended to either repeat FNAB or to conduct molecular testing, molecular imaging, or diagnostic lobectomy, in combination with the diagnostic methods mentioned before [14].

Moreover, the sensitivity and specificity of FNAB are also limited in patients with follicular papillary carcinoma who lack the standard diagnostic criteria (arrangement of cells in papillary formations) or who do not have clear features (nuclear inclusion) [15]. Besides these cases, FNAB is also not significantly effective in patients with small follicular goiter and hypercellular configuration [16]. The majority of nodules are ultimately proven to be benign (55–85%) [17,18,19]. Despite this, for a definitive histopathological diagnosis, surgical exclusion is required in every nodule in which FNAB exhibits follicular findings [12,16], although the majority of them are proven to be benign (55–85%) [17,18,19].

The goal of this review is to highlight the use of molecular imaging with 99mTc-Sestamibi, as a valuable adjunct for evaluating thyroid nodules with inconclusive cytological findings. After minimally invasive techniques for the treatment of thyroid nodules, clinical, biochemical, and US assessments are recommended after 6–12 months as a follow up, while re-evaluation should take place after 3–5 years. In cases of changes in TIRADS category, further follow-up in 3–5 years is recommended, whereas in cases of no further changes after 2 years, the time interval until re-evaluation may be extended.

Just like Polyphemus, the most famous cyclops, FNAB is very powerful within its line of sight and is completely blind beyond.

## 4. The Phaeacians: Scintigraphy 

After a terrible storm, Odysseus found shelter in Phaeacia, where the locals provided answers and help, helping Odysseus finally return to his beloved homeland and family.

Like the Phaeacians, thyroid scintigraphy provides the help and accuracy needed to achieve the ultimate objective—namely, a definitive diagnosis that can lead to the ideal treatment. This is exactly why the American Thyroid Association 2016 guidelines emphasized that in cases of abnormal TSH levels, not only an US, but also a thyroid scintigraphy should also be performed before FNAB to determine the functional status of the nodule [20].

Seven to fifteen percent of thyroid nodules are malignant [3]. The majority of thyroid cancers appear as “cold” on either 99mTc-O4-pertechnetate or radioactive iodine scintigraphy, while “hot” nodules are unlikely to be malignant (<1%) [21]. This reduced radiopharmaceutical uptake is due to the reduction or loss in the sodium/iodine symporter activity in the majority of thyroid cancers [22]. However, a differential diagnostic problem arises as a variety of benign entities might also appear “cold” (e.g., abscess, colloid nodule, and adenoma) [23]. Therefore, new thyroid scintigraphic methods are sought to maximize the accuracy of the result.

99mTc-SESTAMIBI (Sestamibi), also known as 99mTc-methoxy isobutyl isonitrile, is a lipophilic radiopharmaceutical, widely used for functional studies of the heart, parathyroid, and breast tumors. Since the 1990s, myocardial perfusion scintigraphy with Sestamibi has been an important diagnostic tool for nuclear cardiology and the most frequently performed nuclear medicine examination worldwide [24]. Sestamibi is absorbed by cells with mitochondrial fibrillation and negatively charged cell membranes [25]. The negative transmembrane potential allows Sestamibi to passively diffuse into the mitochondria [26] and based on these characteristics, tissues with adequate blood flow and a high metabolic activity show increased uptake and accumulation. A lack of intake is observed in reduced perspiration. This occurs in the absence of mitochondrial activity (e.g., in myocardial infarction) or outflow mechanisms (e.g., in drug-resistant cancers with elevated P-glycoprotein and protein-1 associated with multiple-drug resistance) [27].

Sestamibi, as a lipophilic cation, enters reversibly into the cytoplasm thermodynamically and then passes irreversibly through the mitochondrial membrane to the upper extremity due to the high mitochondrial membrane. Cancer cells, with their large metabolic cycle, are characterized by a higher electrical potential in the mitochondrial membrane, leading to an increased accumulation of Sestamibi compared to normal cells. Therefore, the physical characteristics of Sestamibi make it an ideal non-specific oncophilic radio-detector that can be widely used for the characterization of thyroid nodules [28].

The first thyroid nodule imaging studies with Sestamibi were performed as early as the mid-1990s [29]. In 2013, Treglia et al., evaluating 21 studies, published the first meta-analysis of the effectiveness of Sestamibi scintigraphy in the characterization of thyroid nodules suspected of malignancy [30]. They found that the pooled-sensitivity and pooled-specificity of the thyroid cancer detection method were 85.1% and 45.7% respectively, when tested independently of 99mTc or 123I scintigraphy in hypofunctional nodules. Meanwhile, the sensitivity and specificity of Sestamibi were estimated at 82.1% and 62%, respectively. This variability among the included studies may present a potential bias, as the studies showed statistical differences in their sensitivity and specificity. Systematic reviews combine studies that often differ in clinical characteristics, methodological approaches, sample size, study quality, and inclusion and exclusion criteria. Therefore, an appropriate assessment of heterogeneity, through subgroup analyses or alternative techniques, is essential for drawing more reliable and safer conclusions [30]. The imaging protocols followed by each research group vary in several aspects, including the administered activity of isonitrile, the use of radiotherapy for scintigraphy following Sestamibi imaging, the timing and the type imaging performed, and the characteristics of Single-Photon Emission Computed Tomography with Computed Tomography (SPECT/CT) systems used at each center. Accordingly, differences are found in the evaluation of results (visual, quantitative, and semi-quantitative analysis), although none appear to significantly differ from the others [31,32,33].

The accuracy of the method based on the results of histopathological exposure after thyroidectomy has been studied independently and in comparison with other diagnostic methods. The research team of José Luis Beristain Hernández examined 69 Sestamibi patients and found a sensitivity of 89.28%, specificity of 29.25%, positive prognostic value of 46.29%, and negative prognostic value of 80% for the method [34]. The results of Giovanella et al. (2010) are promising for the effectiveness of the method, as none of the 63 patients with a negative Sestamibi examination were diagnosed with malignancy [32]. Therefore, there was not a single case of a false negative result. Overall, a 100% sensitivity, 88% specificity, 89% accuracy, 27% positive predictive value, and 100% negative predictive value were recorded [32]. MM Sathekge et al. concluded that Sestamibi combined with FNAB and three-phase 99mTc scintigraphy could be valuable tools for the preoperative evaluation of thyroid nodules (specificities of 77%, 40%, and 90%, respectively, for each of the methods) [35].

Several research groups compared Sestamibi with other diagnostic methods. For example, Riazi et al. compared the effectiveness of 7 different methods of evaluating Sestamibi results and found accuracies ranging between 69.46% and 92.21%, providing the impetus for the investigation of the appropriate qualitative and quantitative methods [36]. Furthermore, they compared the effectiveness of Sestamibi with FNAB, finding that FNAB has a sensitivity of 66.66%, a specificity of 100%, a negative predictive value of 95.72%, a positive predictive value of 100%, and an accuracy of 96.06% [36]. In an earlier study, Sathekge’s research group compared Sestamibi, 99mTc, and FNAB and found specificities of 77%, 40%, and 90%, respectively [35]. S Sager et al. examined the potential of preoperative Sestamibi for thyroid nodules compared with 18F-FDG, and found that positron emission tomography scan and a computed tomography scan (PET/CT) imaging was not superior to Sestamibi for the differential diagnosis of malignant neoplasms [37]. In the same year, Giovanella’s group published the results of a comparison of the accuracy of isonitrile scintigraphy and molecular analysis for the detection of mutations associated with thyroid malignancies. According to their conclusions, semiquantitative analysis of Sestamibi should be preferred compared to molecular testing for the differential diagnosis of malignant versus benign thyroid nodules. They concluded that Sestamibi was significantly more accurate (positive likelihood ratio of 4.56 for qualitative analysis and 12.35 for semi-quantitative) compared to molecular testing for mutation detection (positive likelihood ratio 1.74). Therefore, a negative Sestamibi could reliably exclude the presence of malignancy [38].

**Figure 1:**
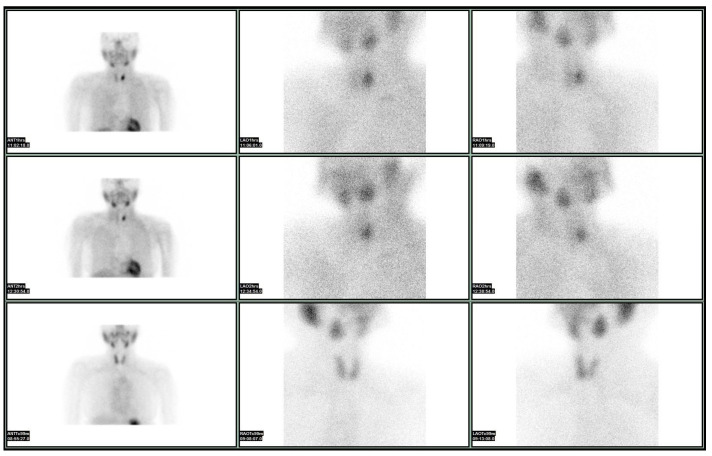
A malignant thyroid nodule with indeterminate cytological features on FNA and a positive SESTAMIBI scan.

In 2012, Leidig-Bruckner et al. studied the effectiveness of diagnostic methods preoperatively characterize nodules [31]. Examining a large sample of patients (N = 391), they found that the sensitivity, specificity, and negative and positive predictive values for Sestamibi were 88.2%, 35.5%, 95.1%, and 17.4%, respectively; for FNAB, they were 38.5%, 90.6%, 90.6%, and 38.5%, respectively; while for the combination of the two 92.3%, 30.6%, 96.3%, and 16.9%, respectively. Therefore, they set the stage for further research on test combinations to reduce the number of unnecessary thyroidectomies [31]. For this reason, Piccardo’s group investigated the role of 18F-FDG nodule imaging, US, and Sestamibi, both independently and in combination, and concluded the following: 18F-FDG detection accuracy of thyroid malignancy is higher than Sestamibi and US. A negative 18F-FDG correctly predicts benign findings in histopathological reports. Positive 18F-FDG and US combination is significantly more specific compared to 18F-FDG alone. Positive 18F-FDG is significantly associated with malignancy, if Sestamibi is negative, which could only be assessed qualitatively [39]. A malignant thyroid nodule exhibiting indeterminate cytological features on fine-needle aspiration and a positive Sestamibi scan (Figure 1).

Therefore, molecular imaging, just like the Phaeacians who provided long-awaited answers and facilitated Odysseus’ return, enables accurate diagnosis, leading clinicians towards proper treatment.

## 5. Ithaca: Treatment

After ten years of relentless struggle against vengeful gods, horrifying monsters, murderous sirens, fierce cyclopes, and treacherous women of astonishing beauty, as well as his companions’ greed and thoughtlessness and his own weaknesses, Odysseus finally returned home to Ithaca as a beggar [40]. But, his adventures were not over yet.

It seems that thyroid cancer patients can also experience prolonged challenges, even after receiving a diagnosis. The quality of life of thyroid cancer patients is worse than that of the general population, as found by Aschebrook-Kilfoy et al. [41] and Pace-Asciak et. al. [42]. It is also worse than that of patients with malignant neoplasms with a worse prognosis, such as colon cancer, breast cancer, gynecological malignancies, and brain gliomas [43]. Singer et. al. [44] noted that QoL may not be directly proportional to the severity of cancer prognosis. According to the systematic review by Hedman et al., thyroidectomy had a significantly negative impact on patients’ Health-Related Quality of Life (HRQoL) in the short term, while HRQoL scores improved as they moved away from the procedure. This was also true for patients whose operation left chronic lesions [45].

In addition to the morbidity and quality of life impact on patients after thyroidectomy, especially for a large-scale operation (including lymph node cleansing), it is particularly burdensome in terms of public health economics and personally for the patient and relatives alike [46]. A number of studies have examined the results of cost−utility analyses of performing thyroidectomies with or without concurrent prophylactic lymphadenectomy. It seems that, from an economic point of view, total thyroidectomy combined with prophylactic lymph node dissection is more expensive both in the medium- and long-term [47]. Other authors have found that routine prophylactic lymph node dissection in patients at low risk for recurrence becomes cost-effective 9 years after surgery, while 20 years later, it can save resources, provided radioactive iodine treatment and pre- and post-operative complications are kept to a minimum [47].

Therefore, in addition to maintaining a high level of quality of life and ensuring a better prognosis for patients, securing health resources seems to be an important concern for researchers in the management of patients with thyroid nodules. Whereas the current trend favors a preference for both minimally invasive and cost-effective diagnostic techniques, e.g., the replacement of diagnostic lobectomies with core needle biopsy, the role of imaging the techniques such as Sestamibi scintigraphy is to provide reliable data to help minimize unnecessary interventions. This role is not fully fulfilled by FNAB, as Trimboli et al. observed that 20% of FNABs are indeterminate, usually involving benign neoplasms (75% of cases) [48]. Therefore, it is important to apply pre-operative, mainly non-invasive methods to characterize thyroid nodules in order to avoid unnecessary surgeries. The high incidence of thyroid nodules and the significance of early and valid differential diagnosis of malignant neoplasms make effective diagnostic methods necessary both for the correct treatment of patients with confirmed malignant thyroid disease and for limiting unnecessary surgical interventions. Ensuring the best prognosis for patients, maintaining a high level of physical well-being and quality of life, but also avoiding high-cost therapeutic interventions that have no indication to be performed, are the goals pursued by the wider use of isonitrile scintigraphy in thyroid nodules of uncertain malignant potential.

## 6. Conclusions

The arrival of Odysseus at Ithaca initially went unnoticed due to his disguise. Revealing his true identity, he began massacring the suitors, who were for years plaguing Penelope, asking for her hand and the kingdom. After killing all 108 of them, he was reunited with his loving wife, son, and father. Finally, catharsis is achieved.

FNAB and Sestamibi are indeed more or less complementary. Other methods, such as US-guided core-needle biopsy and elastography do not significantly limit diagnostic surgeries. So, how can catharsis be achieved in thyroid nodule diagnosis and treatment? Given the fact that, nowadays, Health Policy Decisions are (also) based on cost-effectiveness, new scintigraphic methods providing highly accurate information regarding the presence of malignancy or not should be sought, in order to avoid unnecessary surgeries, overtreatment, and invasive diagnostic techniques.

## Figures and Tables

**Table 1:** Sonographic patterns and the estimated risk of malignancy [8].

Sonographic Pattern	Estimated Risk of Malignancy, %
High suspicion	>70–90
Intermediate suspicion	10–20
Low suspicion	5–10
Very low suspicion	<3
Benign	<1

**Table 2:** The Bethesda system and risk of malignancy [8].

Diagnostic Category	Estimated/Predicted Risk of Malignancy by the Bethesda System, %
Nondiagnostic or unsatisfactory	1–4
Benign	0–3
Atypia of undetermined significance or follicular lesion of undetermined significance	5–15
Follicular neoplasm or suspicious for a follicular neoplasm	15–30
Suspicious for malignancy	60–75
Malignant	97–99

## Data Availability

No new data were created or analyzed in this study. Data sharing is not applicable to this article.

## Abbreviations

The following abbreviations are used in this manuscript:

TSH	Thyroid stimulating hormone
US	Ultrasound
FNAB ACR-TIRADS	Fine needle aspiration biopsy American College of Radiology-Thyroid imaging reporting and data system
TI-RADS	Thyroid imaging reporting and data system
EU-TIRADS	European Thyroid Association TI-RADS
RSS	Risk stratification systems
Sestamibi	99mTc-SESTAMIBI
SPECT-CT	Single-Photon Emission Computed Tomography with Computed Tomography
PET-CT	Positron emission tomography scan and a computed tomography scan

## References

[1] Giannoula E., Melidis C., Frangos S., Papadopoulos N., Koutsouki G., Iakovou I. (2020). Ecological Study on Thyroid Cancer Incidence and Mortality in Association with European Union Member States’ Air Pollution. Int. J. Environ. Res. Public Health.

[2] Dean D.S., Gharib H. (2008). Epidemiology of thyroid nodules. Best Pract. Res. Clin. Endocrinol. Metab..

[3] Wong R., Farrell S.G., Grossmann M. (2018). Thyroid nodules: Diagnosis and management. Med. J. Aust..

[4] Tessler F.N., Middleton W.D., Grant E.G., Hoang J.K., Berland L.L., Teefey S.A., Cronan J.J., Beland M.D., Desser T.S., Frates M.C. (2017). ACR Thyroid Imaging, Reporting and Data System (TI-RADS): White Paper of the ACR TI-RADS Committee. J. Am. Coll. Radiol. JACR.

[5] Russ G., Bonnema S.J., Erdogan M.F., Durante C., Ngu R., Leenhardt L. (2017). European Thyroid Association Guidelines for Ultrasound Malignancy Risk Stratification of Thyroid Nodules in Adults: The EU-TIRADS. Eur. Thyroid J..

[6] Mariani G., Tonacchera M., Grosso M., Fiore E., Falcetta P., Montanelli L., Bagattini B., Vitti P., Strauss H.W. (2021). The Role of Nuclear Medicine in the Clinical Management of Benign Thyroid Disorders, Part 2: Nodular Goiter, Hypothyroidism, and Subacute Thyroiditis. J. Nucl. Med. Off. Publ. Soc. Nucl. Med..

[7] Mistry R., Hillyar C., Nibber A., Sooriyamoorthy T., Kumar N. (2020). Ultrasound Classification of Thyroid Nodules: A Systematic Review. Cureus.

[8] Haugen B.R., Alexander E.K., Bible K.C., Doherty G.M., Mandel S.J., Nikiforov Y.E., Pacini F., Randolph G.W., Sawka A.M., Schlumberger M. (2016). 2015 American Thyroid Association Management Guidelines for Adult Patients with Thyroid Nodules and Differentiated Thyroid Cancer: The American Thyroid Association Guidelines Task Force on Thyroid Nodules and Differentiated Thyroid Cancer. Thyroid.

[9] Rossi E.D., Adeniran A.J., Faquin W.C. (2019). Pitfalls in Thyroid Cytopathology. Surg. Pathol. Clin..

[10] Zhu Y., Song Y., Xu G., Fan Z., Ren W. (2020). Causes of misdiagnoses by thyroid fine-needle aspiration cytology (FNAC): Our experience and a systematic review. Diagn. Pathol..

[11] Parameswaran R., Shulin Hu J., Min En N., Tan W.B., Yuan N.K. (2017). Patterns of metastasis in follicular thyroid carcinoma and the difference between early and delayed presentation. Ann. R. Coll. Surg. Engl..

[12] Rosai J. (2005). Handling of thyroid follicular patterned lesions. Endocr. Pathol..

[13] Cibas E.S., Ali S.Z. (2017). The 2017 Bethesda System for Reporting Thyroid Cytopathology. Thyroid. Off. J. Am. Thyroid Assoc..

[14] Valderrabano P., McIver B. (2017). Evaluation and Management of Indeterminate Thyroid Nodules: The Revolution of Risk Stratification Beyond Cytological Diagnosis. Cancer Control J. Moffitt Cancer Cent..

[15] Liu J., Singh B., Tallini G., Carlson D.L., Katabi N., Shaha A., Tuttle R.M., Ghossein R.A. (2006). Follicular variant of papillary thyroid carcinoma: A clinicopathologic study of a problematic entity. Cancer.

[16] Suster S. (2006). Thyroid tumors with a follicular growth pattern: Problems in differential diagnosis. Arch. Pathol. Lab. Med..

[17] Sahin M., Gursoy A., Tutuncu N.B., Guvener D.N. (2006). Prevalence and prediction of malignancy in cytologically indeterminate thyroid nodules. Clin. Endocrinol..

[18] Trimboli P., Treglia G., Guidobaldi L., Saggiorato E., Nigri G., Crescenzi A., Romanelli F., Orlandi F., Valabrega S., Sadeghi R. (2014). Clinical characteristics as predictors of malignancy in patients with indeterminate thyroid cytology: A meta-analysis. Endocrine.

[19] Castro M.R., Espiritu R.P., Bahn R.S., Henry M.R., Gharib H., Caraballo P.J., Morris J.C. (2011). Predictors of malignancy in patients with cytologically suspicious thyroid nodules. Thyroid Off. J. Am. Thyroid Assoc..

[20] Ross D.S., Burch H.B., Cooper D.S., Greenlee M.C., Laurberg P., Maia A.L., Rivkees S.A., Samuels M., Sosa J.A., Stan M.N. (2016). 2016 American Thyroid Association Guidelines for Diagnosis and Management of Hyperthyroidism and Other Causes of Thyrotoxicosis. Thyroid Off. J. Am. Thyroid Assoc..

[21] Stember J.N., Sheikh A., Perez E., Divgi C., Hamacher K., Jambawalikar S., Yeh R. (2020). A threshold-based method to predict thyroid nodules on scintigraphy scans. Biomed. Phys. Eng. Express.

[22] Meller J., Becker W. (2002). The continuing importance of thyroid scintigraphy in the era of high-resolution ultrasound. Eur. J. Nucl. Med. Mol. Imaging.

[23] Sherman S.I., Gillenwater A.M. (2003). Diagnostic Evaluation of the Solitary Thyroid Nodule. Holland-Frei Cancer Medicine.

[24] Underwood S.R., McCready R., Gnanasegaran G., Bomanji J.B. (2016). A History of Radionuclide Studies in the UK: 50th Anniversary of the British Nuclear Medicine Society.

[25] Márián T., Balkay L., Szabó G., Krasznai Z.T., Hernádi Z., Galuska L., Szabó-Péli J., Esik O., Trón L., Krasznai Z. (2005). Biphasic accumulation kinetics of [99mTc]-hexakis-2-methoxyisobutyl isonitrile in tumour cells and its modulation by lipophilic P-glycoprotein ligands. Eur. J. Pharm. Sci. Off. J. Eur. Fed. Pharm. Sci..

[26] Kawamoto A., Kato T., Shioi T., Okuda J., Kawashima T., Tamaki Y., Niizuma S., Tanada Y., Takemura G., Narazaki M. (2015). Measurement of technetium-99m sestamibi signals in rats administered a mitochondrial uncoupler and in a rat model of heart failure. PLoS ONE.

[27] Rizk T.H., Nagalli S. (2020). Technetium 99m sestamibi. StatPearls.

[28] Bongiovanni M., Paone G., Ceriani L., Pusztaszeri M.J.C., Imaging T. (2013). Cellular and molecular basis for thyroid cancer imaging in nuclear medicine. Clin. Trans. Imaging.

[29] Sundram F.X., Mack P. (1995). Evaluation of thyroid nodules for malignancy using 99Tcm-sestamibi. Nucl. Med. Commun..

[30] Treglia G., Caldarella C., Saggiorato E., Ceriani L., Orlandi F., Salvatori M., Giovanella L. (2013). Diagnostic performance of (99m)Tc-MIBI scan in predicting the malignancy of thyroid nodules: A meta-analysis. Endocrine.

[31] Leidig-Bruckner G., Cichorowski G., Sattler P., Bruckner T., Sattler B. (2012). Evaluation of thyroid nodules--combined use of (99m)Tc-methylisobutylnitrile scintigraphy and aspiration cytology to assess risk of malignancy and stratify patients for surgical or nonsurgical therapy--a retrospective cohort study. Clin. Endocrinol..

[32] Giovanella L., Suriano S., Maffioli M., Ceriani L., Spriano G. (2010). (99m)Tc-sestamibi scanning in thyroid nodules with nondiagnostic cytology. Head Neck.

[33] Hurtado-López L.M., Arellano-Montaño S., Torres-Acosta E.M., Zaldivar-Ramirez F.R., Duarte-Torres R.M., Alonso-De-Ruiz P., Martínez-Duncker I., Martínez-Duncker C. (2004). Combined use of fine-needle aspiration biopsy, MIBI scans and frozen section biopsy offers the best diagnostic accuracy in the assessment of the hypofunctioning solitary thyroid nodule. Eur. J. Nucl. Med. Mol. Imaging.

[34] Beristain Hernández J.L., Servín Torres E., Sosa Caballero A., Velázquez García J.A., Pozzo Bobarín R., Delgadillo Teyer G., Serrano Galeana I., Márquez Hernández A., Bevia Pérez F., Piscil Salazar M.A. (2010). Determination of the diagnostic accuracy of 99mTc sestamibi scanning in patients with thyroid nodule and a definitive histopathological report. Endocrinol. Nutr. Organo Soc. Esp. Endocrinol. Nutr..

[35] Sathekge M.M., Mageza R.B., Muthuphei M.N., Modiba M.C., Clauss R.C. (2001). Evaluation of thyroid nodules with technetium-99m MIBI and technetium-99m pertechnetate. Head Neck.

[36] Riazi A., Kalantarhormozi M., Nabipour I., Eghbali S.S., Farzaneh M., Javadi H., Ostovar A., Seyedabadi M., Assadi M. (2014). Technetium-99m methoxyisobutylisonitrile scintigraphy in the assessment of cold thyroid nodules: Is it time to change the approach to the management of cold thyroid nodules?. Nucl. Med. Commun..

[37] Sager S., Vatankulu B., Erdogan E., Mut S., Teksoz S., Ozturk T., Sonmezoglu K., Kanmaz B. (2015). Comparison of F-18 FDG-PET/CT and Tc-99m MIBI in the preoperative evaluation of cold thyroid nodules in the same patient group. Endocrine.

[38] Giovanella L., Campenni A., Treglia G., Verburg F.A., Trimboli P., Ceriani L., Bongiovanni M. (2016). Molecular imaging with (99m)Tc-MIBI and molecular testing for mutations in differentiating benign from malignant follicular neoplasm: A prospective comparison. Eur. J. Nucl. Med. Mol. Imaging.

[39] Piccardo A., Puntoni M., Treglia G., Foppiani L., Bertagna F., Paparo F., Massollo M., Dib B., Paone G., Arlandini A. (2016). Thyroid nodules with indeterminate cytology: Prospective comparison between 18F-FDG-PET/CT, multiparametric neck ultrasonography, 99mTc-MIBI scintigraphy and histology. Eur. J. Endocrinol..

[40] Psyhoumtakis G., Alexiou S., Makromichelaki Mintza T. (2010). Homer’s Odyssey.

[41] Aschebrook-Kilfoy B., James B., Nagar S., Kaplan S., Seng V., Ahsan H., Angelos P., Kaplan E.L., Guerrero M.A., Kuo J.H. (2015). Risk Factors for Decreased Quality of Life in Thyroid Cancer Survivors: Initial Findings from the North American Thyroid Cancer Survivorship Study. Thyroid Off. J. Am. Thyroid Assoc..

[42] Pace-Asciak P., Russell J.O., Tufano R.P. (2023). Review: Improving quality of life in patients with differentiated thyroid cancer. Front. Oncol..

[43] Hoftijzer H.C., Heemstra K.A., Corssmit E.P., van der Klaauw A.A., Romijn J.A., Smit J.W. (2008). Quality of life in cured patients with differentiated thyroid carcinoma. J. Clin. Endocrinol. Metab..

[44] Singer S., Lincke T., Gamper E., Bhaskaran K., Schreiber S., Hinz A., Schulte T. (2012). Quality of life in patients with thyroid cancer compared with the general population. Thyroid Off. J. Am. Thyroid Assoc..

[45] Hedman C., Djärv T., Strang P., Lundgren C.I. (2017). Effect of Thyroid-Related Symptoms on Long-Term Quality of Life in Patients with Differentiated Thyroid Carcinoma: A Population-Based Study in Sweden. Thyroid Off. J. Am. Thyroid Assoc..

[46] Duan H., Gamper E., Becherer A., Hoffmann M. (2015). Quality of life aspects in the management of thyroid cancer. Oral Oncol..

[47] Lang B.H., Wong C.K. (2014). A cost-minimization analysis comparing total thyroidectomy alone and total thyroidectomy with prophylactic central neck dissection in clinically nodal-negative papillary thyroid carcinoma. Ann. Surg. Oncol..

[48] Trimboli P., Nasrollah N., Amendola S., Crescenzi A., Guidobaldi L., Chiesa C., Maglio R., Nigri G., Pontecorvi A., Romanelli F. (2015). A cost analysis of thyroid core needle biopsy vs. diagnostic surgery. Gland Surg..

